# Biotechnological Conversion of Grape Pomace to Poly(3-hydroxybutyrate) by Moderately Thermophilic Bacterium *Tepidimonas taiwanensis*

**DOI:** 10.3390/bioengineering8100141

**Published:** 2021-10-14

**Authors:** Xenie Kourilova, Iva Pernicova, Michaela Vidlakova, Roman Krejcirik, Katerina Mrazova, Kamila Hrubanova, Vladislav Krzyzanek, Jana Nebesarova, Stanislav Obruca

**Affiliations:** 1 Department of Food Chemistry and Biotechnology, Faculty of Chemistry, Brno University of Technology, Purkynova 118, 612 00 Brno, Czech Republic; xckourilovax@fch.vut.cz (X.K.); xcpernicovai@fch.vut.cz (I.P.); Michaela.Vidlakova@vut.cz (M.V.); xckrejcirik@fch.vut.cz (R.K.); 2 Institute of Scientific Instruments of the Czech Academy of Sciences, v.v.i., Kralovopolska 147, 612 64 Brno, Czech Republic; Katerina.Mrazova@vut.cz (K.M.); hrubanova@isibrno.cz (K.H.); krzyzanek@ISIBrno.Cz (V.K.); 3 Biology Centre, The Czech Academy of Sciences, v.v.i., Branisovska 31, 370 05 Ceske Budejovice, Czech Republic; nebe@paru.cas.cz; 4 Faculty of Science, Charles University, Vinicna 7, 128 43 Prague 2, Czech Republic

**Keywords:** polyhydroxyalkanoates, *Tepidimonas taiwanensis*, grape pomace, thermophiles

## Abstract

Polyhydroxyalkanoates (PHA) are microbial polyesters that have recently come to the forefront of interest due to their biodegradability and production from renewable sources. A potential increase in competitiveness of PHA production process comes with a combination of the use of thermophilic bacteria with the mutual use of waste substrates. In this work, the thermophilic bacterium *Tepidimonas taiwanensis* LMG 22826 was identified as a promising PHA producer. The ability to produce PHA in *T. taiwanensis* was studied both on genotype and phenotype levels. The gene encoding the Class I PHA synthase, a crucial enzyme in PHA synthesis, was detected both by genome database search and by PCR. The microbial culture of *T. taiwanensis* was capable of efficient utilization of glucose and fructose. When cultivated on glucose as the only carbon source at 50 °C, the PHA titers reached up to 3.55 g/L, and PHA content in cell dry mass was 65%. The preference of fructose and glucose opens the possibility to employ *T. taiwanensis* for PHA production on various food wastes rich in these abundant sugars. In this work, PHA production on grape pomace extracts was successfully tested.

## 1. Introduction

Pollution of the environment by solid resistant petrochemical polymers represents one of the most important ecological problems. Every year, several million tons of plastic are produced, which has a wide range of uses, but a large part of them end up being thrown away and accumulating in nature. A potential solution of this problem might be, at least in part, the substitution of petrochemical polymers with biodegradable and renewable materials. One of the alternatives is polyhydroxyalkanoates (PHA). PHA are microbial polyesters that are produced by numerous prokaryotes in the form of intracellular granules to store energy and carbon, but in addition, PHA reveal a protective function concerning the adverse effects of the environment [1]. PHA can be divided based on chain length into scl-PHA (short-chain length), which contains three to five carbons in the monomer, and mcl-PHA (medium-chain length), which contains 6 to 14 carbons in the monomer. A homopolymer of 3-hydroxybutyrate, poly(3-hydroxybutyrate)(PHB) is accumulated by numerous prokaryotes; it can be stated to be the most common and the best-studied member of the PHA family [2,3]. Generally, PHA are a great alternative to petrochemical plastics. They are fully biodegradable and biocompatible; therefore, PHA demonstrate broad application potential [4] in medicine [5] or cosmetics [6], but also in numerous fields of industry, such as the food industry, packaging industry, or agriculture [7]. However, their disadvantage is still the high cost of production as compared to petrochemical polymers. The price of a commercially produced PHA can range from USD 2 to 5 per kilogram, but the price of, for example, polypropylene is about USD 1 per kilogram [8]. The use of waste substrates, stemming especially from the food industry or agriculture, as input carbon sources can reduce the costs. Furthermore, the price of the material can also be influenced by selecting a suitable microbial producer of PHA, for example, from the ranks of extremophilic microorganisms.

Extremophiles are organisms that live and prosper under extreme conditions relative to human life. These are environments with high or very low pH, temperature, pressure, or salinity values [9]. Previously, it was thought that these sites, such as salt lakes, black smokers, or geothermal springs, were dead, but in fact, they are abundantly inhabited by extremophile microorganisms [10]. In recent years, extremophiles have been at the forefront of science and industry. By adapting them to extreme environments, they offer great potential for applications [11]. Their enzymes, also called extremozymes, have wide use in applications where the conditions are unfavorable for “common” enzymes [12]. However, that is not the only advantage of extremophiles in biotechnology. Their unusual cultivation conditions also offer new possibilities for the biotechnology process. As a result, the sterility requirements of the entire production process may be reduced, thereby lowering the cost of biotechnological production and enhancing the competitiveness of the entire biotechnology application. The introduction of extremophiles into the industry is the main idea of the new generation of industrial biotechnology [13].

There are several producers of PHA among extremophiles. Generally, the most studied extremophilic producers of PHA are halophiles [14]. These are microorganisms that thrive in the presence of salt. PHA is not only used to store carbon and energy, but PHA also helps halophiles survive fluctuations in osmotic pressure [15]. Halophilic PHA producers include not only bacteria but also archaea. One of the most famous producers belonging to the Archaea domain is *Haloferax medeterranei* [16]. It is also able to use various waste substrates from the food industry. Another well-studied genus is *Halomonas* [17]. There are many producers such as *Halomonas halophila* [18], *Halomonas boliviensis* [19] *Halomonas campisalis* [20], or *Halomonas hydrothermalis*, which is capable of producing PHA from waste frying oil [21]. However, high salt loads required by halophilic PHA producers bring numerous complications, such as heavy corrosion of equipment, increased medium costs, and demanding waste-water management.

Therefore, a group of thermophilic microorganisms is also of interest with respect to PHA production. These microorganisms thrive at temperatures of 45 °C and above. There are also some described PHA producers in this group, including *Chelatococcus thermostellatus* [22], *Caldimonas taiwanensis* [23], or new isolate bacteria *Aneurinibacillus* sp. H1 [24]. PHA production has also recently been described in the bacterium *Schelegelella thermodepolymerans* using xylose as the most preferred substrate [25].

Grape wine is one of the most popular alcoholic beverages, and its consumption is increasing every year. The annual production of grapes is approximately 78 million tonnes (2018), almost 60% of which are intended for the production of alcoholic beverages [26]. Wine production produces waste in several steps; the most important waste is grape pomace—solid residues after the preparation of grape juice. Grape pomace comprises 10 to 30% of the weight of processed wine and consists mainly of husks, seeds, and any other solids remaining after pressing [27]. It is a complex material; its composition is determined by grape variety, season, weather conditions, etc., but generally, its composition is approximately 30% neutral polysaccharides, 20% acidic pectic substances, 15% insoluble proanthocyanins, lignin, and structural proteins [28]. The profile and quantity of sugars are based on the variety used. Red wines have a lower proportion of soluble sugars that have already been used in fermentation by yeast. White varieties, on the other hand, have a higher proportion of soluble sugars, with a fructose content of almost 9% and glucose of up to 27% per dry mass [29]. Grape pomace has several uses; it can be used as a food supplement [30], fertilizer [31], or feed [32,33,34]. However, it can also be used as a carbon waste source for biotechnology purposes [35,36]. Therefore, this material can be a suitable resource to produce PHA. The use of grape pomace to produce PHA has already been tested by mesophilic producers to reduce the cost of the biotechnology process. For example, *Pseudomonas putida* KT2440 was used to produce mcl-PHA on grape pomace in a two-stage process. The process involved a growth phase on grape pomace extract of the Gewürztraminer variety, and then a mixture of octanoic acid and 10-undecenoic acid was added in the production phase. With this bioengineering approach, the dry biomass mass was about 14 g/L, and the copolymer poly(3-hydroxyoctanoate-*co*-3-hydroxy-10-undecenoate) represented 41% of cell dry mass. The experiment was carried out in a laboratory fermenter and is a promising option to increase the competitiveness of mcl-PHA [36].

Grape pomace extract was also used to produce PHA using *Cupriavidus necator* H16 or halophiles such as *Halomonas halophila* or *Halomonas organivorans*. The prepared lyophilized grape extract contained 33% fructose and 28% glucose. Using 20 g/L of grape sugar extract in the flask test, the cell dry mass of *Cupriavidus necator* H16 reached 4 g/L, and PHA production was almost 2 g/L. Furthermore, PHA production using halophilic strains produced comparable results of 1.8 g/l for *Halomonas halophila*, and the highest production was recorded in *Halomonas organivorans* 2.1 g/L [35]. Extremophiles may be a suitable alternative to mesophiles regarding PHA production. Combined with extremophilic producers, costs may fall, thanks to the use of waste substrates and the reduction in sterility claims, thus causing a decrease in the price of PHA production and an increase in their competitiveness.

The genus *Tepidimonas* accommodates moderately thermophilic Gram-negative, rod-shaped, chemoorganoheterotrophic, motile bacteria with a single polar flagellum. Although these bacteria are not described as PHA producers, they harbor gene machinery for PHA synthesis. However, their biotechnological potential is limited by their relatively low catabolic flexibility. Members of the *Tepidimonas* genus are capable of the utilization of organic acids and amino acids, but usually, carbohydrates are not assimilated. The unique exception is *Tepidimonas taiwanensis*, which should be capable of the utilization of glucose and fructose [37], the main monosaccharides present in grape pomace. Therefore, in this work, *T. taiwanensis* LMG 22826 was evaluated for the first time regarding its PHA synthetic potential. Special attention was paid to its capability to synthesize PHA from inexpensive grape pomace, which could be a route toward sustainable and feasible PHA production.

## 2. Materials and Methods

### 2.1. Microorganisms and Cultivation

The bacterium *Tepidimonas taiwanensis* LMG 22826 was purchased from Belgian coordinate collections of microorganisms, Ghent University. This bacterial strain was cultivated usually for 24 h in complex medium (Nutrient Broth with peptone 1 wt.%) and then for 72 h in mineral medium (Na_2_HPO_4_ · 12 H_2_O (9.0 g/L), KH_2_PO_4_ (1.5 g/L), NH_4_Cl (1.0 g/L), MgSO_4_ · 7 H_2_O (0.2 g/L), CaCl_2_ · 2 H_2_O (0.02 g/L), Fe^(III)^NH_4_citrate (0.0012 g/L), yeast extract (0.5 g/L), 1 mL/L of microelements solution (EDTA (50.0 g/L), FeCl_3_ · 6 H_2_O (13.8 g/L), ZnCl_2_ (0.84 g/L), CuCl_2_ · 2 H_2_O (0.13 g/L), CoCl_2_ · 6 H_2_O (0.1 g/L), MnCl_2_ · 6 H_2_O (0.016 g/L), H_3_BO_3_ (0.1 g/L), dissolved in distilled water), with a carbon source (mainly glucose, 20 g/L) to promote PHA synthesis. In the case of the inoculation phase, the bacteria were cultivated in 100 mL Erlenmeyer flasks with a medium volume of 50 mL. The production part of the cultivation was performed in 250 mL Erlenmeyer flasks with a total volume of 100 mL of medium, including 10 vol.% of inoculum. In both parts of cultivation, Erlenmeyer flasks were constantly shaken at 50 °C unless otherwise noted.

Screening of the optimal carbon source was performed in 96-well microtiter plates. Tested carbon sources were fructose, galactose, glucose, glycerol, lactose, mannose, soluble starch, sucrose, and xylose. Each well was inoculated by 20 µL of inoculum, 75 µL 2× concentrated mineral medium, and 75 µL relevant carbon source (40 g/L). Optical density was measured using ELISA reader at 630 nm, (ELx808, Biotek, Winooski, VA, USA) at the beginning of cultivation and after 48 h. From the difference of the measured values, it was possible to compare the increase in the bacterial culture.

The optimal cultivation temperature was tested under the conditions described at the beginning of Section 2.1. The selected temperatures were 45, 50, 55, and 60 °C. 

Based on the optimized carbon sources and temperature, the ability to produce copolymers was also investigated. Precursors for accumulation of copolymers poly(3-hydroxybutyrate-*co*-3-hydroxyvalerate) (P{3HB-*co*-3HV}) and P(3-hydroxybutyrate-*co*-4-hydroxybytyrate) (P{3HB-*co*-4HB}) were chosen; specifically for 3HV introduction, valeric acid and sodium propionate were applied at a concentration of 2 g/L along with 20 g/L of glucose; for 4HB, 1,4-butanediol and γ-butyrolactone were tested as precursors at a concentration of 8 g/L as the sole carbon source. Medium with 20 g/L of glucose was used as a control.

Based on the obtained data, a suitable waste substrate—grape pomace—was selected. The individual components of the mineral medium were dissolved directly in the grape pomace extract, not in distilled water. Subsequently, cultivations were performed under optimal conditions (50 °C, 180 rpm). In the case of cultivation with diluted extracts, the extracts were diluted with distilled water in a ratio 1:1.

### 2.2. Verification of PHA Production at Genotype and Phenotype Level

Multiplex Polymerase Chain Reaction (PCR) was performed to confirm the ability to produce PHA at the genotype level. The procedure was the same as reported previously [18]. Bacterial DNA (*16SrRNA* gene) and PHA class I synthase (*phaC* gene) were detected. 

PHA content in bacterial biomass was determined in dry bacterial biomass. Gas chromatography with flame ionization detection was used for the analysis. The conditions were set as previously described [38]. Product yield coefficient (Y_P/S_) was calculated, which is determined as a ratio of the amount of product to substrate consumed by microbial culture.

The cell morphology was investigated employing transmission electron microscopy (TEM) analysis, using the microscope JEOL 1010 (JEOL, Tokyo, Japan) as described previously [39]. The cells were centrifuged, and a concentrated pellet was pipetted on 3 mm aluminum carriers pretreated with 1% solution of lecithin in chloroform and fixed using the high-pressure freezer EM ICE (Leica Microsystems, Vienna, Austria). Frozen samples were transferred into freeze substitution unit AFS2 (Leica Microsystems, Vienna, Austria). The substitution solution contained 1.5% OsO_4_ in acetone, and the substitution protocol was set as previously described [40]. Following freeze-substitution, samples were washed with acetone for 15 min 3 times and were gradually infiltrated with mixtures of epoxy resin (Epoxy Embedding Medium kit, Sigma-Aldrich, St. Louis, MO, USA) and acetone in ratios 1:2, 1:1, and 2:1 and pure resin (1 h each). Samples were then left in fresh pure resin overnight under vacuum and finally embedded in fresh pure resin using 62 °C heat for 48 h. Embedded samples were then cut to ultrathin sections using a diamond knife with cutting angle 45 (Diatome, Nidau, Switzerland) and ultramicrotome UTC Ultracut (Leica Microsystems, Vienna, Austria). Sections were stained with uranyl acetate and lead citrate. Additionally, the elastic behavior of the PHA granules, which was previously described [41], was confirmed by cryogenic scanning electron microscopy (cryo-SEM) using the microscope equipped with a cryo stage (Magellan 400/L, FEI). The concentrated pellet of cells was pipetted on 6 mm aluminum carrier and fixed using high-pressure freezing (EM ICE, Leica Microsystems, Vienna, Austria). Frozen samples were transferred into cryo-vacuum preparation chamber (ACE600, Leica Microsystems) where the samples underwent freeze fracturing and sublimation at −95 °C for 7 min. Following sublimation, cells were observed in a scanning electron microscope at −120 °C using 1−2 keV electron beam.

### 2.3. Grape Pomace Extract

The grape pomace used was obtained from Rathouzsky winery from the Moravské Bránice. The extracts were prepared from the following varieties: Muller Thurgau weiss, Veltliner fruehrot, Palava, Sauvignon, Pinot blanc, and Blaufraenkisch. Extracts were obtained by mixing grape pomace with water (5% *w*/*v*). Subsequently, the pH was corrected to 4 with 1 M H_2_SO_4_ and 0.2% (*v*/*v*) of Viscozyme was added. The incubation occurred for 24 h at 50 °C and 100 rpm. Subsequently, the contents were filtered and pH neutralized with 30% (*w*/*v*) NaOH.

The content of monosaccharides, organic acids, and total phenolic substances was determined in the grape pomes extract. In the case of monosaccharides, high-performance liquid chromatography Shimadzu 10AD with a refractive index detector was used for analysis (Shimadzu Corporation, Kyoto, Japan). The separation conditions were as follows: a Waters Carbohydrate Analysis 3.9 × 300 mm column, isocratic elution, mobile phase (acetonitrile: water (80:20)), and flow rate 1.2 mL/min.

Organic acids were determined by ion chromatography 850 Professional, Metrohm with conductivity detector (column—Metrosep Organic Acids, 250/7.8, Metrohm; mobile phase—0.5 mM sulphuric acid and 15% acetone in water, 0.5 mL/min; suppressor—10 mM LiCl).

A slightly modified Folin–Ciocalteu method [42] was used to determine the total phenolic content. First, 1 mL Folin–Ciocalteu reagent diluted 10× was mixed with 1 mL distilled water and 50 μL sample diluted as needed. All components were mixed and incubated for 5 min at room temperature. Subsequently, 1 mL of a saturated sodium carbonate solution was added, mixed, and incubated for 15 min. The absorbance of the mixture was measured at 750 nm using a UV-VIS spectrophotometer (Nanophotometer, Implen P300, Implen GmbH, Munchen, Germany).

## 3. Results and Discussion

### 3.1. PHA-Related Genes of Strain T. taiwanensis LMG 22826

PHA are produced by numerous prokaryotic microorganisms. An important predisposition of PHA production by prokaryotes is the presence of genes encoding a crucial enzyme required for PHA synthesis–PHA synthase (PhaC). These enzymes are divided into four classes based on substrate specificity and subunit composition [43]. The thermophilic bacterium *Tepidimonas taiwanensis* LMG 22826 can be a suitable candidate for PHA production [37]. However, in general, the *Tepidimonas* species are scarcely studied in terms of PHA production. Several genome assemblies of various *T. taiwanensis* strains are available including that of LMG 22826 (also marked as I1-1strain), accession number of this genome assembly in GeneBank is ASM755667v1. Our basic bioinformatics analysis demonstrated that *T. taiwanensis* LMG 22826 harbors enzyme machinery necessary for PHA synthesis; it was found that the bacterium contains the gene encoding for the Class I PHA synthase (Uniprot accession number A0A554X3G4). The encoded protein exhibits some homology with other Class I PHA synthases, primarily with those from other *Proteobacteria* strains, including the genus *Burkholderia*, for which PHA production is already described [44,45]. The genus *Tepidimonas* also belongs to the order *Burkholderiales*. In addition to PHA synthase, the sequence of a protein called a polyhydroxyalkanoate synthesis repressor (PhaR) was also identified (Uniprot accession number A0A554X7W2). The presence of PHA depolymerase (PhaZ), the enzyme responsible for the mobilization of PHA granules is also known from the available genome assemblies (Uniprot accession number A0A554X7W2). 

**Figure 1:**
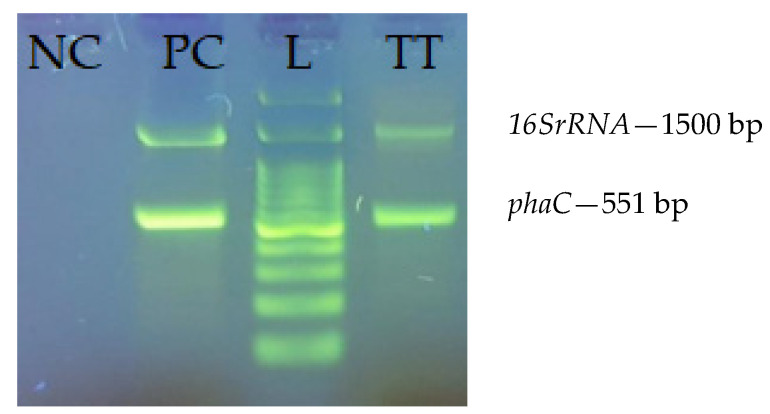
Detection of Class I PHA synthase by conventional PCR. NC—negative control; PC—positive control (*Cupriavidus necator* H16); L—DNA ladder; TT—*Tepidimonas taiwanensis* LMG 22826.

However, in addition to public database screening of genes involved in PHA metabolism, PHA synthase was also verified using the conventional PCR method. When multiplex PCR was used to confirm both bacterial DNA, gene *16SrRNA*, and confirmation of PHA synthase class I. Figure 1 shows confirmation of the presence of Class I PHA synthase gene. *T. taiwanensis* LMG 22826 is therefore well equipped to be a producer of PHA. However, this still needs to be verified on the phenotype level. 

### 3.2. Screening of PHA Production and the Influence of Culture Parameters on PHA Production

**Figure 2:**
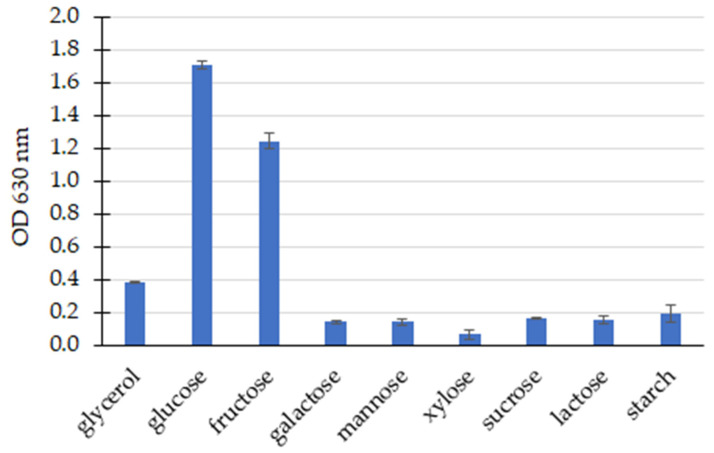
Measured turbidity at OD 630 nm for growth of *T. taiwanensis* biomass for various substrates.

After confirmation of the predisposition to produce PHA on the genotype level, the ability to produce PHA using *T. taiwanensis* LMG 22826 was also verified on the phenotype level. To detect PHA in dry biomass, it was first necessary to ensure sufficient growth of the strain. Therefore, various substances such as glycerol, glucose, fructose, galactose, mannose, xylose, sucrose, lactose, and starch were screened as carbon substrates at 20 g/L (Figure 2). The screening was performed only in a microtitration plate where absorbance was measured. Glucose and fructose proved to be the most suitable substrates, which is in accordance with Chen et al., who also stated that *T. taiwanesis* is capable of the utilization of these sugars as the only carbohydrates [37].

PHA production was further tested in flasks with glucose and fructose as the carbon sources, the presence of PHA was detected by gas chromatography. The biomass and PHA titers reached 5.23 g/L and 3.55 g/L on glucose, respectively. The yields on fructose were slightly lower—about 3.6 g/L of cell dry mass and 2.12 g/L of PHA. According to expectation, the bacterial culture accumulated PHB homopolymer. 

**Figure 3:**
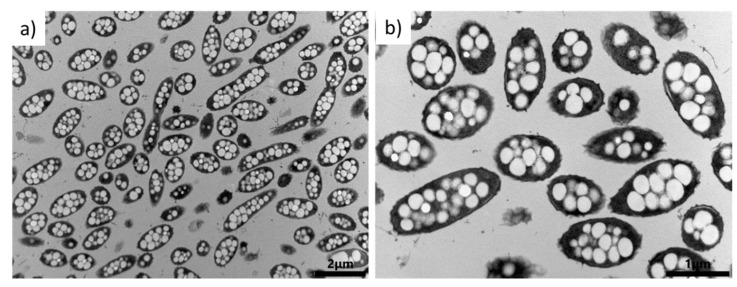
Morphology of PHA and bacterial strain *T. taiwanensis* as seen by TEM. (**a**) lower magnification; (**b**) higher magnification.

**Figure 4:**
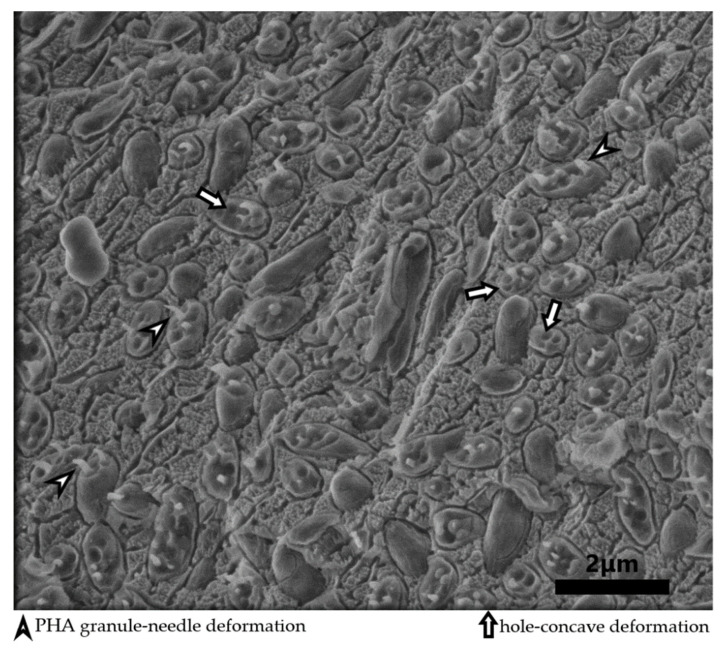
Deformation of PHA granules in the cells of *T. taiwanensis* was caused by freeze fracturing observed using cryo-SEM.

In addition, the morphology of *Tepidimonas taiwanensis* LMG 22826 cells grown on glucose was inspected by TEM analysis. As can be seen in Figure 3, the cells are filled with PHA granules. The number of granules in the cell is rather high, over 10 smaller granules per cell. Generally, the morphology of granules in cells is different and depends on the species of bacteria. For example, *Cupriavidus necator* H16 contains three to seven larger granules in the cell [39], whereas the halophilic bacterium *Halomonas hydrothermalis* contains a large number of small PHA granules in the cell [21]. Additionally, as was previously described for *Cupriavidus necator* H16, PHA granules stay elastic at temperatures of liquid nitrogen and can be observed using freeze fracturing method followed by cryo-SEM [41]. Figure 4 showes cryo-SEM microphotograph of fractured cells of *Tepidimonas taiwanensis* LMG 22826. Needle type of deformation of the PHA granules can be observed, together with concave deformation-holes where the PHA granules were placed before being pulled out of the cell during fracturing.

In order to increase the production of PHA, it is necessary to optimize culture conditions; therefore, the ability to grow and produce PHA in the range of cultivation temperatures 45 to 60 °C (with a step-up of 5 °C) was also tested. The obtained data (Table 1) indicate that the bacterium can grow throughout the range of temperatures tested. However, the highest biomass and PHA productions were obtained at temperatures of 50 and 55 °C. The temperature of 50 °C was selected for further experiments when the cell dry mass (CDM) concentration was 5.45 g/L and the PHB concentration was 3.6 g/L. Ibrahim and Steinbuchel proved that even a temperature of 50 °C is high enough to prevent fermentation from contamination and that the process of PHA production can be operated for long periods in semi-sterile mode [46].

*T. taiwanensis* proved to be a suitable producer of PHA, especially PHB homopolymer. Therefore, we also tested its ability to synthesize various copolymers using precursors of 3-hydroxyvalerate or 4-hydroxybutyrate. Generally, the copolymers reveal superior mechanical and technological properties as compared to PHB homopolymer. PHB itself is relatively crystalline and therefore brittle and unflexible, but copolymers containing 3HV have a lower crystalline content [47]. Moreover, copolymers with 4HB are even more elastic and have wider applications in medical applications [48]. Nevertheless, *T. taiwanensis* was unable to produce a copolymer with 4HB (see Table 2). However, it was able to form a P(3HB-*co*-3HV) copolymer using the sodium propionate precursor. When the total PHA content was 44% by weight of dry biomass, resulting in PHA titers of 1.8 g/L, the 3HV content in the copolymer was 6.6 mol. %.

### 3.3. Use of Grape Pomace to Produce PHA

#### 3.3.1. Characterization of Extract of Grape Pomace

Grapes are generally one of the most cultivated crops in the world. They are not only intended for direct consumption but also the production of other foods (jam, wine vinegar, grape oil, dried kernel extracts, etc.) [49]. One of the most popular products is wine. The processing of grapes into wine produces a variety of wastes, including grape pomace. Thus, grape pomace represents a significant part of the waste from the winery. They have some other uses, especially recently developing their use as potential resources for added value [50]. Their composition depends on the variety used and the procedure of wine preparation. Grape pomace can also be made into a sugar-rich extract, which can be used by microbiological conversion to PHA, for example.

Grape pomace extracts were prepared as described in Section 2.3, and they were analyzed before being utilized as substrates for PHA production employing *T. taiwanensis*. Organic acids (citric acid, malic acid, lactic acid, acetic acid, formic acid, propionic acid), total polyphenols, sugars, mainly glucose and fructose, were analyzed in the extracts. As can be seen in Table 3, the parameters monitored vary among the varieties used. The highest content of organic acids was determined in Veltliner fruehrot extract, and the highest concentrations of total polyphenols were determined in Blaufraenkisch extract (red), where the highest sugar content was also determined. 

Generally, grape pomace extracts have a wide application, depending on the wine variety and the preparation of the extract. For example, red wine extracts were found to contain higher levels of flavonoid and phenolic compounds and exhibit stronger oxygen radical absorption capabilities than apple pomace. Grape pomace extracts also indicate potential medical use in the treatment of diabetes [51], and they are also used in natural cosmetic products [52].

#### 3.3.2. Grape Pomace Extracts as a Carbon Source for PHA Production

The ability to produce PHA has been confirmed in *Tepidimonas taiwanensis*, and it was also confirmed that the bacterium is capable of using both glucose and fructose. Since glucose and fructose comprise the dominant portion of sugars in the prepared extracts of wine pomace, these extracts were used to produce PHA. Extracts without dilution supplemented by the components of the cultivation media were tested first; the results are demonstrated in Table 4. The grape pomace extract of the variety of Veltliner fruehrot demonstrated the highest potential since CDM of the bacterial culture reached 4.36 g/L and PHA titer was 2.09 g/L. High PHA yields were also achieved on grape pomace extracts of Sauvignon (1.498 g/L PHA) and Pinot blanc (1.585 g/L). It is important to note that, for these extracts, the yield coefficient Y_P/S_ was almost identical (0.22), but the highest yield coefficient was obtained for Blaufraenkisch (rose), but even though the use of sugars was effective, the PHA content was low and was only around 12% by CDM.

With respect to PHA production from grape pomace extract, *T. taiwanensis* can be considered being very promising bacterium. In a similar study, Kovalcik et al. obtained comparable or lower PHB titers employing *Halomonas halophila* (1.8 g/L of PHB), *Halomonas organivorans* (2.1 g/L of PHB) and *Cupriavidus necator* (1.9 g/L PHB) [35]. Nevertheless, *T. taiwanesis* provides all the above-mentioned advantages associated with the cultivation of thermophilic bacterium.

*T. taiwanensis* was shown to be capable of producing PHA on grape pomace extract. Nevertheless, apart from desirable sugars, the extracts also contain phenolic substances, organic acids a and also other substances that can have an inhibitory effect for microbial culture [53]. To minimize inhibition of the culture by substances naturally present in grape pomace extracts, the extracts were diluted 1:1 with distilled water, supplemented with all the constituents of the production medium and used for PHA production employing *T. taiwanesis*. This dilution resulted in the potential dilution of inhibitory substances and, unfortunately, also total sugars. Generally, PHA is overproduced by microbial cultures when carbon source is present in excess and other elements (e.g., sources of nitrogen, phosphorous, etc.) are limiting. Nevertheless, there are reports that even high excess of carbon source has a negative impact on PHA accumulation. Shang et al. reported that the highest amount of PHB was accumulated by Ralstonia eutropha (former designation for Cupriavidus necator) when cultivated on 9 g/L of glucose; when glucose concentration was increased, the PHB yields decreased significantly [54]. In Table 5, the effect of the diluted medium on the production and growth of the bacterium can be observed. It is noticeable that, for some extracts, dilution had a positive effect. For example, for the Palava extract, 2.59 g/l of biomass was achieved (in undiluted only 0.8 g/L) and PHA production also increased from the original 0.03 g/L to 0.6 g/l. However, some extracts also had a negative effect, with the production of Vetliner fruehrot extract, for example, falling from an initial 2.090 g/L to 0.883 g/L. This decrease may be due to the lack of a carbon source due to the dilution of the extracts, because, at the end of the culture, no residual sugars were detected in any use of the diluted extract. This may indicate a carbon limitation, which is not very suitable for PHA production. However, the production of PHA employing *T. taiwanensis* on waste sources such as grape pomace extracts is a suitable alternative to the production of PHA due to the extremophilic nature of the producer, and because the use of waste as an input source increases the competitiveness of the production.

## 4. Conclusions

Based on the obtained results, it was proved that the thermophilic bacterium *Tepidimonas taiwanensis* LMG 22826 possesses gene machinery for PHA biosynthesis, and it is a suitable candidate for PHA production, taking advantage of its thermophilic nature. The optimal cultivation temperature of this bacterium is in the range of 50–55 °C, which substantially reduces the risk of contamination by common mesophilic microflora, and hence reduces the sterility demands of the process. It was also found that this bacterium is capable of efficient utilization of glucose and fructose; therefore, it can be employed for thermophilic production of PHA on food-waste substrates rich in these abundant sugars. In this work, we utilized *T. taiwanensis* for PHA production on grape pomace—the major waste stream of wine production, which is produced annually in extensive amounts. PHA titers on Veltliner fruehrot extract in flasks cultivation reached 2.09 g/L, which is more than comparable to PHA production reported to yields reported in the literature for mesophilic bacteria. Therefore, *T. taiwanensis* is a suitable candidate for the biotechnological production of PHA.

## Figures and Tables

**Table 1:** Effect of culture temperature on PHA production using glucose as the sole source of carbon.

Temperature (°C)	CDM (g/L)	P(3HB) (%)	PHB (g/L)
45	5.08 ± 0.11	67.3 ± 2.1	3.417 ± 0.076
50	5.45 ± 0.22	65.5 ± 5.7	3.559 ± 0.169
55	5.36 ± 0.16	63.9 ± 2.9	3.421 ± 0.056
60	1.60 ± 0.08	49.8 ± 8.5	0.801 ± 0.178

**Table 2:** Ability to produce copolymers containing 3HV or 4HB from different types of precursors.

Precursors	CDM (g/L)	P(3HB) (%)	PHA (g/L)	3HV (mol. %)	4HB (mol. %)
control	4.21 ± 0.19	49.2 ± 4.1	2.069 ± 0.041	n.d.	n.d.
1,4-butanediol	0.32 ± 0.12	14.2 ± 2.5	0.091 ± 0.022	n.d.	n.d.
γ-butyrolactone	0.08 ± 0.01	n.d	n.d.	n.d.	n.d.
valeric acid	0.03 ± 0.01	n.d	n.d.	n.d.	n.d.
sodium propionate	4.16 ± 0.17	44.8 ± 3.8	1.860 ± 0.130	6.6	n.d.

n.d.—not detected.

**Table 3:** Characterization of grape pomace.

Composition of Grape Pomace Extracts		Muller Thurgau weiss	Veltliner Fruehrot	Palava	Sauvignon	Pinot Blanc	Blaufraenkisch (Red)	Blaufraenkisch (Rose)
Organic acid (g/L)	Citric acid	0.009	0.096	0.027	0.147	0.137	0.017	0.104
	Malic acid	0.620	0.939	0.956	0.979	0.0849	0.319	0.877
	Succinic acid	0.093	0.054	0.045	0.101	0.115	0.107	0.012
	Lactic acid	n.d.	0.006	0.006	0.009	0.014	0.022	0.001
	Acetic acid	0.131	0.156	0.059	0.100	0.119	0.071	0.054
	Formic acid	n.d.	0.004	0005	0.004	0.006	0.004	n.d.
	Propionic acid	0.062	0.067	0.064	0.064	0.066	0.061	0.052
Total polyphenols (g/L)		0.453	0.301	0.520	0.160	0.231	0.821	0.548
Sugars (g/L)	Fructose	5.49	4.73	6.68	2.84	2.97	11.65	9.04
	Glucose	5.71	6.61	6.66	4.13	4.31	11.09	8.22
Total sugars (g/L)		11.20	11.35	13.34	6.97	7.28	22.73	17.26

n.d.—not detected.

**Table 4:** PHA production using grape pomace as a carbon source.

Grape Pomace From	CDM (g/L)	P(3HB) (%)	P(3HB) (g/L)	Residual Fructose (g/L)	Residual Glucose (g/L)	Y_P/S_
Muller Thurgau weiss	0.35 ± 0.02	1.4 ± 0.8	0.005 ± 0.001	4.65	4.66	0.00
Veltliner fruehrot	4.36 ± 0.04	47.9 ± 2.3	2.090 ± 0.081	0.74	0.38	0.21
Palava	0.80 ± 0.04	3.7 ± 1.2	0.030 ± 0.001	6.06	6.11	0.03
Sauvignon	3.37 ± 0.13	44.5 ± 1.7	1.498 ± 0.041	0.05	n.d.	0.22
Pinot blanc	3.62 ± 0.01	43.8 ± 2.5	1.585 ± 0.046	0.12	n.d.	0.22
Blaufraenkisch (red)	0.27 ± 0.03	8.4 ± 2.6	0.022 ± 0.001	9.06	8.69	0.00
Blaufraenkisch (rose)	1.92 ± 0.35	12.3 ± 1.9	0.236 ± 0.049	8.62	8.04	0.39

n.d.—not detected.

**Table 5:** Production of PHA using diluted grape spread with water in a 1:1 ratio.

Grape Pomace From	CDM [g/L]	P(3HB) [%]	P(3HB) [g/L]	Residual Fructose [g/L]	Residual Glucose [g/L]	Y_P/S_
Muller Thurgau weiss	1.09 ± 0.24	11.4 ± 1.2	0.115 ± 0.013	n.d.	n.d.	0.02
Veltliner fruehrot	2.39 ± 0.12	36.9 ± 2.4	0.883 ± 0.044	n.d.	n.d.	0.16
Palava	2.59 ± 0.05	31.2 ± 3.4	0.619 ± 0.070	n.d.	n.d.	0.09
Sauvignon	1.32 ± 0.08	27.2 ± 1.7	0.359 ± 0.054	n.d.	n.d.	0.10
Pinot blanc	1.97 ± 0.06	28.6 ± 5.2	0.564 ± 0.013	n.d.	n.d.	0.15
Blaufraenkisch (red)	2.87 ± 0.81	41.9 ± 3.7	1.201 ± 0.076	n.d.	n.d.	0.11
Blaufraenkisch (rose)	2.94 ± 0.09	47.7 ± 2.5	1.399 ± 0.041	n.d.	n.d.	0.16

n.d.—not detected.

## References

[1] Lim H., Chuah J., Chek M., Tan H., Hakoshima T., Sudesh K. (2021). Identification of regions affecting enzyme activity, substrate binding, dimer stabilization and polyhydroxyalkanoate (PHA) granule morphology in the PHA synthase of *Aquitalea* sp. USM4. Int. J. Biol. Macromol..

[2] Raza Z., Abid S., Banat I. (2018). Polyhydroxyalkanoates: Characteristics, production, recent developments and applications. Int. Biodeterior. Biodegrad..

[3] Peña C., Castillo T., García A., Millán M., Segura D. (2014). Biotechnological strategies to improve production of microbial poly-(3-hydroxybutyrate): A review of recent research work. Microb. Biotechnol..

[4] Yadav B., Talan A., Tyagi R., Drogui P. (2021). Concomitant production of value-added products with polyhydroxyalkanoate (PHA) synthesis: A review. Bioresour. Technol..

[5] Ray S., Kalia V. (2017). Biomedical Applications of Polyhydroxyalkanoates. Indian J. Microbiol..

[6] Chen G., Patel M. (2012). Plastics Derived from Biological Sources: Present and Future. Chem. Rev..

[7] Poltronieri P., Kumar P., Martínez L., Kharissova O., Kharisov B. (2017). Polyhydroxyalcanoates (PHAs) in Industrial Applications. Handbook of Ecomaterials.

[8] Crutchik D., Franchi O., Caminos L., Jeison D., Belmonte M., Pedrouso A., Val del Rio A., Mosquera-Corral A., Campos J. (2020). Polyhydroxyalkanoates (PHAs) Production: A Feasible Economic Option for the Treatment of Sewage Sludge in Municipal Wastewater Treatment Plants?. Water.

[9] Rampelotto P. (2013). Extremophiles and Extreme Environments. Life.

[10] Berlemont R., Gerday C. (2011). Extremophiles. Comprehensive Biotechnology.

[11] Irwin J. (2020). Overview of extremophiles and their food and medical applications. Physiol. Biotechnol. Asp. Extrem..

[12] Dumorne K., Cordova D., Astorga-Elo M., Renganathan P. (2017). Extremozymes: A Potential Source for Industrial Applications. J. Microbiol. Biotechnol..

[13] Chen G., Jiang X. (2018). Next generation industrial biotechnology based on extremophilic bacteria. Curr. Opin. Biotechnol..

[14] Quillaguamán J., Guzmán H., Van-Thuoc D., Hatti-Kaul R. (2010). Synthesis and production of polyhydroxyalkanoates by halophiles: Current potential and future prospects. Appl. Microbiol. Biotechnol..

[15] Sedlacek P., Slaninova E., Koller M., Nebesarova J., Marova I., Krzyzanek V., Obruca S. (2019). PHA granules help bacterial cells to preserve cell integrity when exposed to sudden osmotic imbalances. New Biotechnol..

[16] Alsafadi D., Al-Mashaqbeh O. (2017). A one-stage cultivation process for the production of poly-3-(hydroxybutyrate-co-hydroxyvalerate) from olive mill wastewater by Haloferax mediterranei. New Biotechnol..

[17] Tan D., Wu Q., Chen J., Chen G. (2014). Engineering Halomonas TD01 for the low-cost production of polyhydroxyalkanoates. Metab. Eng..

[18] Kucera D., Pernicová I., Kovalcik A., Koller M., Mullerova L., Sedlacek P., Mravec F., Nebesarova J., Kalina M., Marova I. (2018). Characterization of the promising poly(3-hydroxybutyrate) producing halophilic bacterium Halomonas halophila. Bioresour. Technol..

[19] Rivera-Terceros P., Tito-Claros E., Torrico S., Carballo S., Van-Thuoc D., Quillaguamán J. (2015). Production of poly(3-hydroxybutyrate) by Halomonas boliviensis in an air-lift reactor. J. Biol. Res.-Thessalon..

[20] Kulkarni S., Kanekar P., Nilegaonkar S., Sarnaik S., Jog J. (2010). Production and characterization of a biodegradable poly (hydroxybutyrate-co-hydroxyvalerate) (PHB-co-PHV) copolymer by moderately haloalkalitolerant Halomonas campisalis MCM B-1027 isolated from Lonar Lake, India. Bioresour. Technol..

[21] Pernicova I., Kucera D., Nebesarova J., Kalina M., Novackova I., Koller M., Obruca S. (2019). Production of polyhydroxyalkanoates on waste frying oil employing selected Halomonas strains. Bioresour. Technol..

[22] Ibrahim M., Willems A., Steinbüchel A. (2010). Isolation and characterization of new poly(3HB)-accumulating star-shaped cell-aggregates-forming thermophilic bacteria. J. Appl. Microbiol..

[23] Sheu D., Chen W., Yang J., Chang R. (2009). Thermophilic bacterium Caldimonas taiwanensis produces poly(3-hydroxybutyrate-co-3-hydroxyvalerate) from starch and valerate as carbon sources. Enzym. Microb. Technol..

[24] Pernicova I., Novackova I., Sedlacek P., Kourilova X., Kalina M., Kovalcik A., Koller M., Nebesarova J., Krzyzanek V., Hrubanova K. (2020). Introducing the Newly Isolated Bacterium *Aneurinibacillus* sp. H1 as an Auspicious Thermophilic Producer of Various Polyhydroxyalkanoates (PHA) Copolymers–1. Isolation and Characterization of the Bacterium. Polymers.

[25] Kourilova X., Pernicova I., Sedlar K., Musilova J., Sedlacek P., Kalina M., Koller M., Obruca S. (2020). Production of polyhydroxyalkanoates (PHA) by a thermophilic strain of Schlegelella thermodepolymerans from xylose rich substrates. Bioresour. Technol..

[26] (2020). 2019 Statistical Report on World Vitiviniculture, 1st ed.; International Organisation of Vine and Wine Intergovernmental Organisation: International Organisation of Vine and Wine Intergovernmental Organisation. http://oiv.int/public/medias/6782/oiv-2019-statistical-report-on-world-vitiviniculture.pdf.

[27] Moreno A., Ballesteros M., Negro M. (2020). Biorefineries for the valorization of food processing waste. The Interaction of Food Industry and Environment.

[28] Dávila I., Robles E., Egüés I., Labidi J., Gullón P. (2017). The Biorefinery Concept for the Industrial Valorization of Grape Processing By-Products. Handbook of Grape Processing By-Products.

[29] Antonić B., Jančíková S., Dordević D., Tremlová B. (2020). Grape Pomace Valorization: A Systematic Review and Meta-Analysis. Foods.

[30] González-Paramás A., Esteban-Ruano S., Santos-Buelga C., de Pascual-Teresa S., Rivas-Gonzalo J. (2004). Flavanol Content and Antioxidant Activity in Winery Byproducts. J. Agric. Food Chem..

[31] Manios T. (2004). The composting potential of different organic solid wastes: Experience from the island of Crete. Environ. Int..

[32] Sánchez A., Ysunza F., Beltrán-García M., Esqueda M. (2002). Biodegradation of Viticulture Wastes by Pleurotus: A Source of Microbial and Human Food and Its Potential Use in Animal Feeding. J. Agric. Food Chem..

[33] Zacharof M. (2017). Grape Winery Waste as Feedstock for Bioconversions: Applying the Biorefinery Concept. Waste Biomass Valorization.

[34] Taurino R., Ferretti D., Cattani L., Bozzoli F., Bondioli F. (2019). Lightweight clay bricks manufactured by using locally available wine industry waste. J. Build. Eng..

[35] Kovalcik A., Pernicova I., Obruca S., Szotkowski M., Enev V., Kalina M., Marova I. (2020). Grape winery waste as a promising feedstock for the production of polyhydroxyalkanoates and other value-added products. Food Bioprod. Process..

[36] Follonier S. (2015). Pilot-scale Production of Functionalized mcl-PHA from Grape Pomace Supplemented with Fatty Acids. Chem. Biochem. Eng. Q..

[37] Chen T., Chou Y., Chen W., Arun B., Young C. (2006). *Tepidimonas taiwanensis* sp. nov., a novel alkaline-protease-producing bacterium isolated from a hot spring. Extremophiles.

[38] Obruca S., Petrik S., Benesova P., Svoboda Z., Eremka L., Marova I. (2014). Utilization of oil extracted from spent coffee grounds for sustainable production of polyhydroxyalkanoates. Appl. Microbiol. Biotechnol..

[39] Mravec F., Obruca S., Krzyzanek V., Sedlacek P., Hrubanova K., Samek O., Kucera D., Benesova P., Nebesarova J., Steinbüchel A. (2016). Accumulation of PHA granules in Cupriavidus necator as seen by confocal fluorescence microscopy. FEMS Microbiol. Lett..

[40] Kouřilová X., Schwarzerová J., Pernicová I., Sedlář K., Mrázová K., Krzyžánek V., Nebesářová J., Obruča S. (2021). The First Insight into Polyhydroxyalkanoates Accumulation in Multi-Extremophilic Rubrobacter xylanophilus and Rubrobacter spartanus. Microorganisms.

[41] Obruca S., Sedlacek P., Krzyzanek V., Mravec F., Hrubanova K., Samek O., Kucera D., Benesova P., Marova I., Chen G.-Q. (2016). Accumulation of Poly(3-hydroxybutyrate) Helps Bacterial Cells to Survive Freezing. PLoS ONE.

[42] Singleton V., Rossi J. (1965). Colorimetry of Total Phenolics with Phosphomolybdic-Phosphotungstic Acid Reagents. Am. J. Enol. Eiticulture.

[43] Tan I., Foong C., Tan H., Lim H., Zain N., Tan Y., Hoh C., Sudesh K. (2020). Polyhydroxyalkanoate (PHA) synthase genes and PHA-associated gene clusters in *Pseudomonas* spp. and *Janthinobacterium* spp. isolated from Antarctica. J. Biotechnol..

[44] Pan W., Perrotta J., Stipanovic A., Nomura C., Nakas J. (2012). Production of polyhydroxyalkanoates by Burkholderia cepacia ATCC 17759 using a detoxified sugar maple hemicellulosic hydrolysate. J. Ind. Microbiol. Biotechnol..

[45] Kourmentza C., Costa J., Azevedo Z., Servin C., Grandfils C., De Freitas V., Reis M. (2018). Burkholderia thailandensis as a microbial cell factory for the bioconversion of used cooking oil to polyhydroxyalkanoates and rhamnolipids. Bioresour. Technol..

[46] Ibrahim M., Steinbüchel A. (2010). High-Cell-Density Cyclic Fed-Batch Fermentation of a Poly(3-Hydroxybutyrate)-Accumulating Thermophile, *Chelatococcus* sp. Strain MW10. Appl. Environ. Microbiol..

[47] Keenan T., Nakas J., Tanenbaum S. (2006). Polyhydroxyalkanoate copolymers from forest biomass. J. Ind. Microbiol. Biotechnol..

[48] Norhafini H., Huong K., Amirul A. (2019). High PHA density fed-batch cultivation strategies for 4HB-rich P(3HB-co-4HB) copolymer production by transformant Cupriavidus malaysiensis USMAA1020. Int. J. Biol. Macromol..

[49] Venkitasamy C., Zhao L., Zhang R., Pan Z. (2019). Grapes. Integrated Processing Technologies for Food and Agricultural By-Products.

[50] Chowdhary P., Gupta A., Gnansounou E., Pandey A., Chaturvedi P. (2021). Current trends and possibilities for exploitation of Grape pomace as a potential source for value addition. Environ. Pollut..

[51] Hogan S., Zhang L., Li J., Sun S., Canning C., Zhou K. (2010). Antioxidant rich grape pomace extract suppresses postprandial hyperglycemia in diabetic mice by specifically inhibiting alpha-glucosidase. Nutr. Metab..

[52] Pinto D., Cádiz-Gurrea M., Silva A., Delerue-Matos C., Rodrigues F. (2021). Cosmetics—food waste recovery. Food Waste Recovery.

[53] Luchian C., Cotea V., Vlase L., Toiu A., Colibaba L., Răschip I., Nadăş G., Gheldiu A., Tuchiluş C., Rotaru L. (2019). Antioxidant and antimicrobial effects of grape pomace extracts. BIO Web Conf..

[54] Shang L., Jiang M., Chang H.N. (2003). P oly(3-hydroxybutyrate) synthesis in fed-batch culture of Ralstonia eutropha with phosphate limitation under different glucose concentrations. Biotechnol. Lett..

